# Antibiotic prescribing in general medical and surgical specialties: a prospective cohort study

**DOI:** 10.1186/s13756-019-0603-6

**Published:** 2019-09-13

**Authors:** E. Charani, E. de Barra, T. M. Rawson, D. Gill, M. Gilchrist, N. R. Naylor, A. H. Holmes

**Affiliations:** 1 NIHR Health Protection Research Unit in Healthcare Associated Infections and Antimicrobial Resistance, Hammersmith Campus, Imperial College London, 8th Floor Commonwealth Building, Du Cane Road, London, W12 ONN UK; 2 Royal College of Surgeons in Ireland, RCSI Education & Research Centre, Beaumont Hospital, Beaumont, Dublin 9, Ireland; 3 Department of Biostatistics and Epidemiology, School of Public Health, Imperial College London, London, W2 1PG UK; 4 Department of Pharmacy, Imperial College Healthcare NHS Trust, St Mary’s Hospital, Praed Street, London, W12 1NY UK

**Keywords:** Antibiotic-decision making, Surgery, Antibiotic escalation

## Abstract

**Background:**

Qualitative work has described the differences in prescribing practice across medical and surgical specialties. This study aimed to understand if specialty impacts quantitative measures of prescribing practice.

**Methods:**

We prospectively analysed the antibiotic prescribing across general medical and surgical teams for acutely admitted patients. Over a 12-month period (June 2016 – May 2017) 659 patients (362 medical, 297 surgical) were followed for the duration of their hospital stay. Antibiotic prescribing across these cohorts was assessed using Chi-squared or Wilcoxon rank-sum, depending on normality of data. The t-test was used to compare age and length of stay. A logistic regression model was used to predict escalation of antibiotic therapy.

**Results:**

Surgical patients were younger (*p* < 0.001) with lower Charlson Comorbidity Index scores (*p* < 0.001). Antibiotics were prescribed for 45% (162/362) medical and 55% (164/297) surgical patients. Microbiological results were available for 26% (42/164) medical and 29% (48/162) surgical patients, of which 55% (23/42) and 48% (23/48) were positive respectively. There was no difference in the spectrum of antibiotics prescribed between surgery and medicine (*p* = 0.507). In surgery antibiotics were 1) prescribed more frequently (*p* = 0.001); 2) for longer (*p* = 0.016); 3) more likely to be escalated (*p* = 0.004); 4) less likely to be compliant with local policy (*p* < 0.001) than medicine.

**Conclusions:**

Across both specialties, microbiology investigation results are not adequately used to diagnose infections and optimise their management. There is significant variation in antibiotic decision-making (including escalation patterns) between general surgical and medical teams. Antibiotic stewardship interventions targeting surgical specialties need to go beyond surgical prophylaxis. It is critical to focus on of review the patients initiated on therapeutic antibiotics in surgical specialties to ensure that escalation and continuation of therapy is justified.

## Background

Evidence suggests that a large proportion of antibiotic prescribing in hospitals may be inappropriate [1–3]. Optimising antibiotic prescribing and reducing the use of broad-spectrum agents has been shown to reduce the occurrence of healthcare associated infections (HCAI) in hospitals [4]. The bulk of antibiotic decision-making in hospitals takes place with no direct input from infectious disease or medical microbiology experts. There are many branches of specialism in medical practice [5] with one of the most important being between surgery and medicine. The different cultures and team dynamics across the medical and surgical specialties have been recently described using qualitative methodology [6]. Evidence suggests that antibiotic prescribing in surgery lacks clarity and occurs in the context of disjointed information [6, 7]. Studies from operating room practices have highlighted how the environment and context can influence surgical outcomes [8]. In the case of surgical prophylaxis, teams have reported that though aware of guidelines and policy, they attribute a low priority to policy adherence [9]. Whilst qualitative research is critical for defining the context in which medical decision-making occurs across different specialties, and describes how and why clinicians make antibiotic prescribing decisions, it does not address what the effect of these contextual differences are on clinical practice. In this study we set out to investigate antibiotic prescribing in medical and surgical specialties. Detailing and understanding the real-time clinical patterns of prescribing is critical to the co- design of effective antibiotic optimisation interventions. Though the conditions for which antibiotics are prescribed may differ across specialties, the principles of infection diagnosis and management are the same. This study set out to investigate whether there was significant variation in the antibiotic prescribing practices between surgical and medical teams. This knowledge is critical in order to shift from a one size fits all approach to one that recognizes the specific challenges facing clinicians treating infections across different specialties.

## Methods

### Study sample size calculation

This study was conducted across general medical and general surgical teams in one of the five hospitals of a university affiliated Healthcare NHS Organisation, in London. The hospital selected had the largest Emergency Department (ED) and the highest annual turnover of patients. The hospitals have an established, multidisciplinary antimicrobial stewardship programme (ASP). Studies across the hospitals have previously demonstrated a 10% difference in compliance to the local antibiotic policy between medical and surgical teams, with medical teams demonstrating a higher rate of concordance [10].

A power calculation for two independent study groups with a dichotomous primary endpoint was carried out [11]. Using point prevalence data, anticipated incidence of compliance to policy was estimated to be 83% in surgery and 93% in medicine [10]. Setting the type one error rate to 0.05, and type two error rate to 0.2, and thus achieving a statistical power of 0.8, it was calculated that the study would require 165 patients in each arm.

### Exclusion and inclusion criteria

Between June 2016 – May 2017 adult patients (aged ≥18 years) admitted to the medical and surgical teams and prescribed empiric antibiotics other than for prophylaxis were eligible to be included in the study. Data were collected from each ward on alternate weeks. Patients were prospectively identified on qualitative observations of acute ward rounds as part of a mixed methods study [6, 7]. Patients with a hospital stay less than 24 h, and those from (or transferred to) other specialties were excluded.

### Data collection

This study had a prospective cohort design. The reference group were the medical cohort and the comparison group were the surgical cohort. An accurate assessment of the appropriateness of the prescribed antibiotics requires real-time infectious diseases consultations in parallel to the care provided by the respective teams. In the absence of being able to measure appropriateness, the primary outcome measures selected in this study were compliance to the local antibiotic policy, and changes to antibiotic prescriptions (e.g. escalation or de-escalation). The secondary outcome measures were, duration of antibiotic prescriptions, length of hospital stay, use of microbiology and radiology to guide therapy, and 30-day readmission. Patient demographics, antibiotic prescriptions and corresponding indications, and inflammatory marker results were obtained from the patient notes. One researcher (EC) collected all the data. A single reading of white cell count, C-reactive protein, and temperature, closest to the time of initiating antibiotic therapy were recorded. Procalcitonin is not routinely tested for patients in this hospital. The International Classification of Diseases, tenth revision (ICD-10) coding was used to calculate the age-adjusted Charlson comorbidity index (ACCI) for subjects [12]. Comorbidity was ranked by using the final CCI. The CCI score was ranked as: a) 0–2 low; b) 3–4 moderate; and c) 5 or above, severe [13].

The antibiotic exposure days were measured as the number of days that a patient received one or more systemic antibiotics. The cumulative antibiotics days, where multiple antibiotics prescribed on the same day were counted as multiple antibiotic days, was also measured. Cumulative antibiotic exposure days has been used previously [14], regarding its association with *Clostridium difficile* infection.

Antibiotics were ranked in ascending order according to their relative activity against drug-resistant organisms [15](Table 1).

**Table 1:** Antibiotic ranking

Rank 1	Narrow spectrum, including first-generation and second –generation cephalosporins, amoxicillin, co-trimoxazole, metronidazole, and oral vancomycin;
Rank 2	Broad spectrum, including flouroquinolones, macrolides, third-generation cephalosporins, co-amoxiclav, clindamycin;
Rank 3	Extended spectrum, including antipseudomonal penicillins, antipseudomonal carbapenems and intravenous vancomycin;
Rank 4	Restricted, including colistin, tigecycline, linezolid, and daptomycin

Changes made to prescribed antibiotics were classified, using published criteria [16]. Escalation of therapy was defined as the switch to or addition of an agent with a broader spectrum, or additional coverage, or switch from oral to intravenous therapy. De-escalation of therapy was defined as stopping therapy, or de-escalation from intravenous to oral, or changing to an agent with a less broad-spectrum coverage. If antibiotic choice was restricted or changed to broad spectrum agents due to an allergy to a class or individual antibiotic(s), the therapy was classified as unchanged e.g. not de-escalated or escalated.

The following criteria were used to assess whether the antibiotics prescribed were compliant with local empiric policy:

- Was the indication documented on the electronic chart or the medical notes?
- Did the indication have a matching recommendation in the policy?
- If there was no recommendation in the policy, was there any infection team input?

For cases where the indication was missing or vague e.g. ‘for infection’, this variable was labelled as unclear. The reason for having an unclear category as opposed to missing data was to identify cases where the antibiotic prescription had been prescribed and no indication recorded in the notes or the medication chart. One of the requirements of the policy is that for all therapeutic courses of antibiotics, there should be an indication recorded. If antibiotic therapy did not match the recommendation in the policy for the recorded indication, and there was no infection team input, it was labelled as non-compliant.

### Data analysis

The analysis was carried out using Stata, (StataCorp, College Station, Texas, US) version 13.1. Variation in the descriptive variables between the cohorts was assessed using Chi- squared or Wilcoxon rank-sum, depending on normality of data. An independent t-test was used to determine if there were statistical differences in the age and length of stay of patients in the cohorts. We created a logistic regression model to understand the predictors of antibiotic prescribing (as measured by escalation) and see whether there were differences between the two groups after adjusting for these. Escalation was the measure used in the model as deviations from policy can often have a legitimate reason, and hence compliance to policy was not deemed an appropriate measure of the quality of antibiotic prescribing. The variables specialty, length of stay, ACCI, positive chest X-ray and microbiological results were selected as predictors based on existing literature and discussions with the clinicians [6]. The length of stay, antibiotic exposure days, and the comorbidity index were fitted as categorical variables in the model, as they were skewed in their distributions. Statistical significance was defined as a *p* ≤ 0.05. The length of stay and ACCI were tested in the regression model as both continuous and categorical variables. In both cases, the scaling (i.e. variable) type with the best fit was categorical. In the final analysis, these two variables were fitted as categorical.

## Results

### Descriptive statistics

**Fig. 1:**
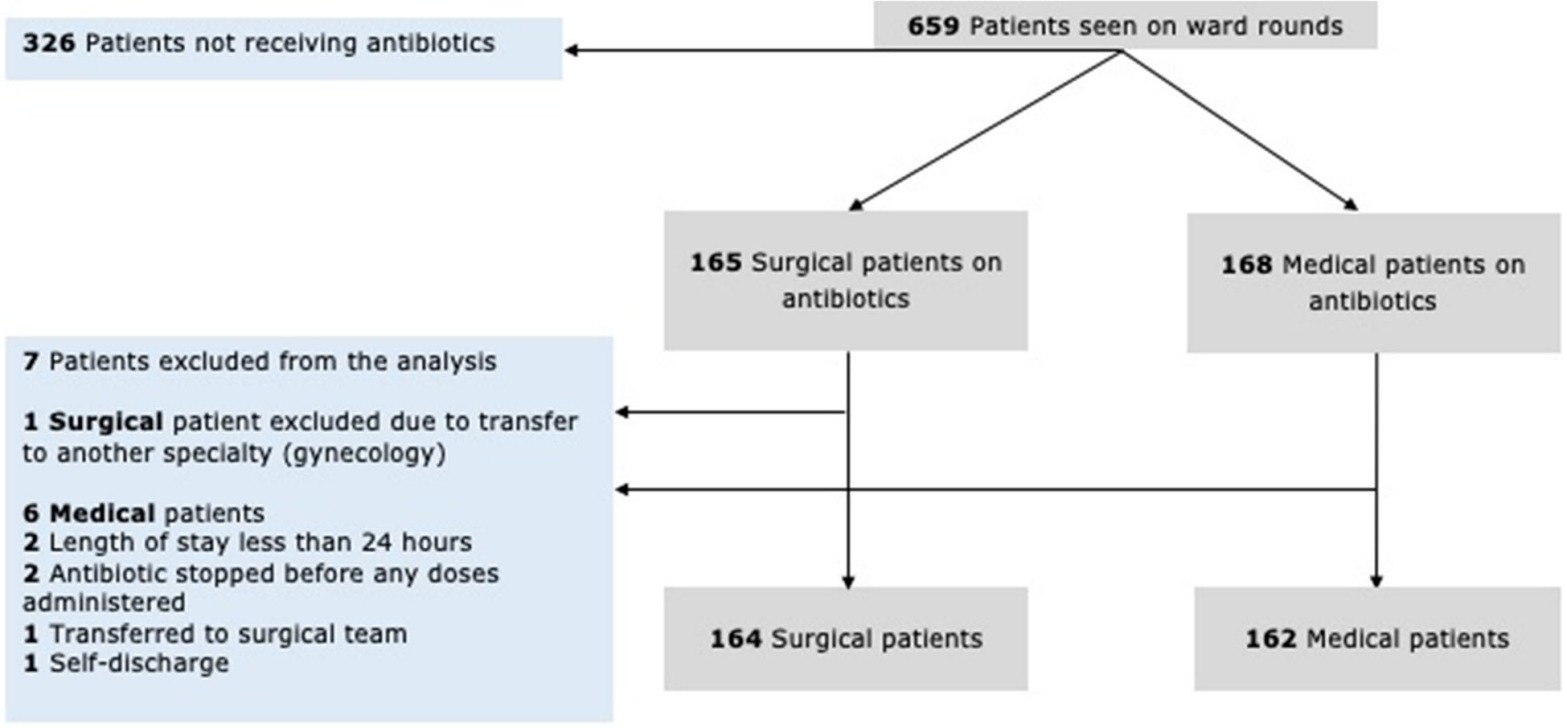
Flow diagram of sample derivation for inclusion in the study

All subjects had complete data for the variables included in this study. The teams admitted 659 patients (362 in medicine and 297 in surgery) during the study period (Fig. 1). One surgical and 6 medical patients were excluded from the statistical analysis in this study (reasons detailed in Fig. 1). Of the included patients, 162 (45%) in medicine, and 164 (55%) in surgery were receiving at least one course of therapeutic antibiotics. During the ward rounds observed in the study, prescribed antibiotics were reviewed in 143/162 (88%) of medical and 111/164 (68%) of surgical patients.

At the time of initiating antibiotics, a normal white cell count was present in 76/162 (47%) of medical, and 52/164 (32%) of surgical patients (Table 2). Antibiotics were started in the absence of a) fever and b) abnormal white blood cell count in 32/162 (19%) of medical and 32/164 (19%) of surgical patients. C-reactive protein was recorded as high, or rising in 308/326 (95%) patients across both specialties. Radiological data in the form of chest x-ray, was available for 90/162 (55%) medical and in 64/164 (39%) surgical patients. In medicine, 36/162 (22%), and in surgery 17/164 (10%) patients had infection confirmed in the radiological report accompanying the chest X-ray. Of all the patients receiving antibiotics 23/162 (14%) in medicine and 23/164 (14%) in surgery had a positive microbiological sample.

**Table 2:** Relative frequency of diagnostic information for patients started on antibiotics in medicine, and surgery (normal ranges are those defined in the local laboratory)

Diagnostic Information	Total 326 *n* (%)	Medicine 162 *n* (%)	Surgery 164 *n* (%)
White Cell Count			
Normal (4.2–11.2 × 10^9^/L)	128 (39.3)	76 (47.0)	52 (31.7)
Abnormal	197 (60.4)	86 (53.0)	111 (68.1)
No Data	1 (0.3)	0	1 (0.6)
Body Temperature			
Normal (36.5–37.5 °C)	168 (51.5)	70 (43.2)	98 (60.0)
Abnormal	157 (48.2)	91 (56.2)	66 (40.0)
No Data	1 (0.3)	1 (0.6)	0
Body temperature and White Cell Count			
Normal	64 (19.4)	32 (19.4)	32 (19.4)
Abnormal	260 (80.0)	129 (80.0)	131(80.0)
No Data	2 (0.6)	1 (0.6)	1 (0.6)
C-reactive Protein			
Normal (< 6 mg/L)	17 (5.2)	11 (7.0)	6 (3.7)
Abnormal (> 6 mg/L)	308 (94.5)	151 (93.0)	157 (95.7)
No Data	1 (0.3)	0	1 (0.6)
Chest X-ray at start of antibiotic			
Yes	154 (47.0)	90 (55.6)	64 (39.0)
No	172 (53.0)	72 (44.4)	100 (61.0)
Sign of infection on chest X-ray			
Yes	53 (16.0)	36 (22.0)	17 (10.0)
No	101 (31.0)	54 (33.3)	47 (29.0)
No imaging	172 (53.0)	72 (44.4)	100 (61.0)
Microbiological culture collected before antibiotic initiation			
Yes	90 (27.6)	42 (26.0)	48 (29.0)
No	236 (72.4)	120 (74.0)	116 (71.0)
The result of microbiological culture collected before antibiotic initiation			
No growth (negative culture)	44 (13.5)	19 (11.7)	25 (15.0)
Pathogen grown (positive culture)	46 (14.1)	23 (14.2)	23 (14.0)
No Culture	236 (72.4)	120 (74.1)	116 (71.0)

There were 395 antibiotic prescriptions in medicine and 461 in surgery. These included the same antibiotic given by a different route or on different occasions to the same patient in the same admission spell. Of the prescribed antibiotic courses, 200/395 (51%) of medical, and 395/461 (86%) surgical were intravenous. Over half, 90/162 (56%) of medical patients were initiated on antibiotics for a respiratory infection (Table 3). Urinary tract infections were the second most common indication in medical patients (29/162; 18%). In surgery, intraperitoneal infections e.g. diverticulitis and appendicitis, were the most common indication (44/164; 27%), followed by biliary infections e.g. cholecystitis (39/164; 24%). Antibiotics were initiated for suspected sepsis in 14/164 (9%) surgical and 5/162 (3%) medical patients. There was no statistically significant difference (*p* = 0.507) in the spectrum and rank of antibiotics that the medical and surgical patients were exposed to during their stay.

**Table 3:** Indication for empiric antibiotic course by specialty (* included non-defined infections in surgery and of various organ systems in medicine)

Indication for antibiotic therapy	Total 326 *n* (%)	Medicine 162 *n* (%)	Surgery 164 *n* (%)
Respiratory	104 (31.90)	90 (55.56)	14 (8.54)
Urinary tract	39 (11.96)	29 (17.90)	10 (6.10)
Intraperitoneal/ Gastrointestinal	48 (14.72)	4 (2.47)	44 (26.83)
Skin and soft tissue	16 (4.91)	8 (4.94)	8 (4.88)
Sepsis	19 (5.83)	5 (3.09)	14 (8.54)
Biliary	39 (11.96)	0	39 (23.78)
Other*	61 (18.71)	26 (16.05)	35 (21.34)

The mean length of stay was 11 days (±1.21) in medicine, 16 days (±1.87) in surgery, t (324), *p* = 0.0553 (Table 3). Medical patients had a significantly higher CCI score (*p* < 0.001). Thirty-day readmission to hospital was significantly higher in medicine versus surgery (*p* < 0.001). There was no difference (*p* = 0.977) in mortality during the admission episode in the two groups. Monotherapy, was more likely (p < 0.001) in medicine than surgery, with 36/162 (22%) vs. 12/164 (7%), of patients being treated with one course of antibiotics during their stay, respectively **(**Table 4**)**. Surgical patients had 50/164 (30%) cumulative antibiotics days equal to or greater than 15, compared with 22/162 (15%) medical patients (*p* = 0.016). Escalation of the initial antibiotic therapy was more likely in the surgical patients than in medical patients, with 49/164 (30%) therapy escalations in surgery compared with 31/162 (19%) in medicine (*p* = 0.0037).

**Table 4:** Univariable analysis of demographic outcomes by specialty

Variable	Measure	Medicine *N* = 162	Surgery *N* = 164	*P* value*
Gender - Male	*n* (%)	76 (47)	84 (51)	0.441
Age at admission	Mean (SD)	70 (18)	55 (22)	–
	Median (Interquartile range)	74 (27–93)	55 (18–92)	< 0.001
	Range (Minimum – Maximum)	21–104	18–95	–
Length of Stay	Mean (SD)	11 (15)	16 (24)	0.0553
	Median (Interquartile range)	7 (1–76)	5 (2–106)	< 0.001
	Range (Minimum – Maximum)	1–95	24–160	–
Charlson comorbidity index (age adjusted)	*n* (%)			
	Low (score of 0–2)	30 (19)	89 (54)	< 0.001
	Moderate (score of 3–4)	39 (24)	23 (14)	
	Severe (score of ≥5)	93 (57)	52 (32)	
30 day readmission	*n* (%)	43 (27)	29 (18)	< 0.001
Mortality in admission episode	*n* (%)	10 (6)	10 (6)	0.977

*Abbreviations*: *n* count, *SD* standard deviation

*P values calculated using Pearson Chi-Squared test or t-test where appropriate.

The lack of a documented indication for prescribed antibiotics made it difficult to assess the compliance to policy for 21/164 (13%) patients in surgery. When included as a three-category variable (Yes/No/Unclear) there was a statistically significant difference (*p* < 0.001) in compliance to policy between medicine (131/162, 80.86) and surgery (108/164, 65.85) (Table 5). In addition to receiving antibiotics during their stay, 80/162 (49%) medical and 73/164 (45%) surgical patients were subsequently discharged home with a course of antibiotics.

**Table 5:** Univariable analysis of antibiotic related variables by specialty

	Medicine 162 *n* (%)	Surgery 164 *n* (%)	*p* value *
Total number of courses of antibiotics	395	461	–
Route of antibiotics (as a total of all courses)			
Intravenous	200 (51)	395 (86)	< 0.001
Oral	195(49)	66 (14)	
Number of antibiotics during spell			
1	36 (22.22)	12 (7.32)	0.001
2	52 (32.10)	68 (41.46)	
≥ 3	74 (45.68)	84 (51.22)	
Antibiotic exposure days			
1–3 days	65 (40)	56 (34)	0.134
4–7 days	55 (34)	52 (32)	
7–14 days	28 (17)	33 (20)	
≥ 15	14 (9)	23 (14)	
Cumulative antibiotic days			
1–7 days	94 (58)	70 (43)	0.016
8–14 days	46 (28)	44 (27)	
≥ 15	22 (14)	50 (30)	
Antibiotic rank			
Narrow	11(6.79)	6 (3.66)	0.507
Broad	98 (60.49)	95 (57.93)	
Extended	47 (29.00)	56 (34.15)	
Restricted	6 (3.70)	7 (4.27)	
Change to initial antibiotic course			
No change/discontinued	76 (46.91)	90 (54.88)	0.0037
Antibiotic escalated	31 (19.14)	49 (29.88)	
Antibiotic de-escalated	55(33.95)	25 (15.24)	
Antibiotic compliant to local policy			
Yes	131 (80.86)	108 (65.85)	< 0.001
No	30 (18.52)	35 (21.34)	
Unclear	1 (0.62)	21 (12.80)	
Antibiotic compliant to local policy (‘unclear’ treated as missing)			
Yes	131 (81.37)	108 (75.52)	0.215
No	30 (18.63)	35 (24.48)	
Antibiotic prescribed on discharge			
Yes	80 (49)	73 (45)	0.677
No	67 (41)	74 (45)	
No data/Patient died	15 (9)	17 (10)	

*P values calculated using Pearson Chi-Squared test or Fisher’s exact test

In the logistic regression model, the unadjusted odds ratio of therapy escalation in surgery versus medicine was 1.801, 95% CI 1.071–3.028, *P* = 0.024. In surgery, 64/164 (39%) of patients underwent an operation during their stay. Having an operation (reference set as not having an operation) was associated with increased odds of escalation of antibiotic therapy (OR = 1.69, 95% CI 0.939–3.042, *p* = 0.082) in the surgical cohort, although this was not statistically significant. In the fully adjusted logistic regression model the odds ratio of antibiotic therapy escalation in surgical patients remained significantly higher compared to medicine (OR = 1.94, 95% CI: 1.07–3.53, *p* = 0.03). Comorbidity, culture and sensitivity results and signs of infection on chest X-ray did not significantly affect the odds of therapy escalation in the regression model. There was a statistically significant association between length of stay and escalation of therapy (Table 6).

**Table 6:** Multiple logistic regression examining the association between escalating antibiotic therapy and the selected variables, adjusting for specialty (n = 326)

	OR	95% CI	Crude P	Adjusted OR^a^	95% CI	Adjusted *P* value
Specialty						
Medicine	1			1		
Surgery	1.801	1.076–3.013	0.024	1.942	1.067–3.534	0.030
Comorbidity score						
Low	1			1		
Mod	1.164	0.629 –	0.629	0.845	0.402–1.773	0.655
Severe	1.380	2.151 0.741–2.570	0.310	1.123	0.541–2.354	0.747
Culture and sensitivity						
Negative	1			1		
Positive	0.569	0.236 –	0.209	0.583	0.218–1.559	0.282
No culture	0.378	1.371 0.192–0.746	0.005	0.470	0.219–1.009	0.053
Los						
1–7	1			1		
8–14	2.027	0.998 –	0.051	2.012	0.962–4.215	0.064
≥ 15	5.758	4.117 3.137–10.567	< 0.001	5.138	2.66–9.910	< 0.001
Sign of infection on chest X-ray						
No	1			1		
Yes	1.145	0.563 –	0.709	1.405	0.635–3.109	0.402
No chest X-ray	0.486	2.323 0.273–0.863	0.014	0.610	0.328–1.173	0.142

^a^ Adjusted for all other variables included within this table

## Discussion

In this study we investigated real-time antibiotic prescribing practices in surgical and medical teams, in order to understand the variation in practice and identify the current gaps. This understanding of the variation in practice across the specialties and teams is necessary, in order to develop bespoke stewardship interventions that target the gaps in practice within specialties. Half the patients received at least one course of antibiotics during their hospital stay. Surgical patients were more likely to receive multiple courses of antibiotics during their hospital stay, and were less likely to have their antibiotic therapy reviewed during ward rounds. The propensity in surgery in this study was to initially prescribe cefuroxime and metronidazole and then step up to intravenous broad-spectrum agents e.g. piperacillin/ tazobactam, and co-amoxiclav with addition of other agents such as gentamicin and vancomycin. In medicine, broad-spectrum agents were more likely to be initiated upon admission and the focus stewardship efforts should be in the initial phase of the patients’ hospital stay. Over the course of the hospital stay however, this study found no statistical difference between the spectrum of antibiotics prescribed in surgery and medicine. This is a new and valuable finding as in surgery, most ASP interventions target surgical prophylaxis or surgical site infection prevention [17, 18]. In surgery stewardship efforts should be focused on patients who remain in hospital in the post-operative phase where they are likely to be initiated on broad spectrum antibiotics.

Diagnostic tests, though available were not routinely used to rationalise antibiotic prescribing. Two of the key interventions in optimising antibiotic use are obtaining a microbiological culture prior to initiation of antibiotic therapy and ensuring relevant imaging [4, 19]. One in five of the patients in both specialties were initiated on antibiotics in the absence of fever and raised white cell count, and culture and sensitivity data were only collected from one third of patients initiated on antibiotics. These findings are consistent with studies reporting inappropriate antibiotic use in hospitals and the inadequate use of available diagnostic tests [15, 20–22]. Identifying the causative organism for the infection, through use of laboratory diagnostics, has been shown to be highly effective in optimising antibiotic therapy [23, 24]. Over 95% of all patients across medicine and surgery had a raised C-reactive protein level at initiation of antibiotic therapy. Published studies have described the role of C-reactive protein in the diagnosis of infections [25, 26], in particular its overuse post-operatively. In post-operative patients C-reactive protein levels can become elevated [26] in response to the trauma of the surgery, and as such the utility of using this marker as a specific marker of infection is inappropriate. Likewise, in medicine the level may be raised due to acute conditions other than infection, such as a myocardial infarct [27–29]. In clinical practice, it is not the absolute C-reactive protein level that should be used, but its trend over time. Large studies on the response of C-reactive protein to antibiotic therapy in infections are lacking [30]. Much work is needed to shift the attitude of healthcare professionals away from their dependency to use C-reactive protein elevation to initiate antibiotics. Clinicians should be encouraged to use their clinical skills for diagnosing infections and to consult with the available pharmacy and infection specialists. Failing that, the routine use of C-reactive protein as a diagnostic marker should be limited.

The surgical patients were primarily receiving intravenous antibiotics. As most of the caseload was intra-abdominal infections the intravenous route is entirely acceptable, especially in patients who have had an operation, and who may have poor gut absorption. Despite these dispensations, only 40% of the surgical patients underwent an operation, however 86% of the antibiotic courses in surgery were intravenous. It would be valuable to repeat this study amongst different surgical specialties to see if the use of intravenous route is as prevalent across the different disciplines in surgery. This study found an association between increased length of stay and antibiotic escalation. Several studies have demonstrated an association between reduced length of hospital stay and de-escalation of antibiotic use, suggesting that de-escalation can lead to reduced length of stay [31, 32]. The reverse can also be expected, that patients who have their antibiotic therapy escalated can be more ill and therefore in need of remaining in hospital. Antibiotic prescribing in the surgical team was also more likely to be non-complaint to local policy and guidelines. The medical specialty were more consistent in providing a rationale and indication for prescribed antibiotics. Including the unclear category in the analysis was important to illustrate the lack of documented indication for antibiotic therapy in surgery. The local policy stipulates that an indication must be documented for prescribed antibiotics. A missing indication could also be classed as inappropriate and therefore non-compliant to the local policy. Including the ‘unclear’ category in the analysis accommodated for this deviation from policy.

This study was conducted alongside an in-depth qualitative ethnographic research that described the contextual differences in decision-making between the teams [6, 7] and identified key differences in how these teams prioritise antibiotic decision-making. The qualitative research reported a model of individualism in surgery, where surgeons driven by performance metrics, are less willing to tolerate uncertainty in antibiotic decision-making [6]. The qualitative findings add value and provide contextual insights to better understand the quantitatively measured antibiotic decision-making behaviours, describe in this cohort study. Practicing a defensive prescribing approach in surgery, and less comfortable with uncertainty in the decisions made for the patients, surgeons may try to avert the risk of a patient developing infections by prescribing broad-spectrum antibiotics. Additionally, the qualitative findings identified the surgical teams to be willing to delegate antibiotic decision-making to the junior staff or other specialties [6, 7]. This can be leveraged to develop better collaborations between stewardship and surgical teams to ensure that antibiotic prescribing in surgery can be optimised.

There are several key recommendations from this study that can be used to develop bespoke stewardship interventions. Antibiotic stewardship interventions in surgery should focus on the post-operative period or in patients who are under surgical care for longer durations, as they are more likely to be on more broad-spectrum antibiotics and to receive multiple courses. Across both specialties more focus needs to be placed on ensuring the correct microbiology tests are performed prior to initiating antibiotic therapy. This can help support correct diagnosis earlier in medial patients to a more targeted spectrum of therapies, and in surgical patients in can help teams with being able to correctly diagnose infections and provide an indication for the prescribed antibiotics.

### Limitations

This is a single-center study focusing on two general medical and surgical teams. The data may therefore not be representative of the breadth of hospital specialties. The cohorts of general medicine and surgery may not be comparable in terms of infection etiology. To address this, one of the primary outcome measures was compliance to policy. This adjusted for the variation between the cohorts, as regardless of the cause of infection the antibiotic therapy would be expected to the compliant to the local policy recommendation for the indication. Since the sample size required to address the research questions was small it was not feasible to include a broad number of infections in the analysis. The inaccuracies of ICD-10 coding have been reported [33]. Any inaccuracies in the coding will have an impact on the ACCI score derived for the patients in this study. For the multivariate regression analysis performed, independent variables that were not available through the data collection methods used, such as infection markers at end of antibiotic therapy could affect prescribing outcomes that were not included in this model. The aforementioned model only includes chest X-ray as a radiological measure. If all the forms of potential diagnostic tests were included, there would be too many categories to consider, for which missing data would be high across different infection-types, and therefore we utilized the most commonly performed diagnostic test.

## Conclusion

One in five patients (across surgery and medicine) were prescribed antibiotics in the absence of raised white cell count or a fever. There is no difference in the spectrum of antibiotics prescribed between medicine and surgery. Surgical patients are significantly more likely to a) receive a greater number of courses of antibiotics; b) have their initial therapy escalated; and c) be on a course not in line with local policy. There are opportunities to widen the reach of ASP and focus on perioperative care, focusing on the review of antibiotic prescriptions in the post-operative phase.

## Data Availability

Data cannot be shared publicly because of confidentiality. Data are available from the Imperial College Institutional Data Access / Ethics Committee (contact via Esmita Charani) for researchers who meet the criteria for access to confidential data.

## Abbreviations

ASP: Antimicrobial stewardship programmes
CCI: Charlson comorbidity index
CI: Confidence interval
ED: Emergency department
OR: Odds ratio
